# Characterization and Manipulation of Carbon Precursor Species during Plasma Enhanced Chemical Vapor Deposition of Graphene

**DOI:** 10.3390/nano10112235

**Published:** 2020-11-11

**Authors:** Otto Zietz, Samuel Olson, Brendan Coyne, Yilian Liu, Jun Jiao

**Affiliations:** 1 Department of Mechanical and Materials Engineering, Portland State University, 1930 SW 4th Ave, Portland, OR 97201, USA; otto@pdx.edu (O.Z.); samolson@pdx.edu (S.O.); bdcoyne@gmail.com (B.C.); 2 Department of Physics, Reed College, 3203 SE Woodstock Blvd., Portland, OR 97202, USA; liub@reed.edu

**Keywords:** graphene, plasma enhanced chemical vapor deposition, low temperature graphene synthesis

## Abstract

To develop a synthesis technique providing enhanced control of graphene film quality and uniformity, a systematic characterization and manipulation of hydrocarbon precursors generated during plasma enhanced chemical vapor deposition of graphene is presented. Remote ionization of acetylene is observed to generate a variety of neutral and ionized hydrocarbon precursors, while in situ manipulation of the size and reactivity of carbon species permitted to interact with the growth catalyst enables control of the resultant graphene morphology. Selective screening of high energy hydrocarbon ions coupled with a multistage bias growth regime results in the production of 90% few-to-monolayer graphene on 50 nm Ni/Cu alloy catalysts at 500 °C. Additionally, synthesis with low power secondary ionization processes is performed and reveals further control during the growth, enabling a 50% reduction in average defect densities throughout the film. Mass spectrometry and UV-Vis spectroscopy monitoring of the reaction environment in conjunction with Raman characterization of the synthesized graphene films facilitates correlation of the carbon species permitted to reach the catalyst surface to the ultimate quality, layer number, and uniformity of the graphene film. These findings reveal a robust technique to control graphene synthesis pathways during plasma enhanced chemical vapor deposition.

## 1. Introduction

Currently, most high quality, large area graphene is produced via chemical vapor deposition (CVD) techniques with gaseous hydrocarbon precursors, micrometer scale Cu as a catalyst and support, and synthesis temperatures in excess of 900 °C [1,2,3,4,5]. Due to the relative thickness and composition of the catalyst and elevated synthesis temperatures, these growths require a transfer process to the target substrate which limits incorporation of graphene to applications with only planar geometries. In recent years, significant research efforts have focused on reducing required synthesis temperatures and catalyst thicknesses with an ultimate goal of developing techniques for direct synthesis on substrates other than transition metal catalysts [6,7,8,9]. The research endeavors for the development of these synthesis techniques aim at eliminating damage and geometry related constraints associated with a transfer process while enabling direct incorporation of graphene in a variety of fields; from the semiconductor industry as an ultrathin diffusion barrier to the aerospace industry as lightweight strengthening and protective coatings [10,11,12,13]. Researchers have identified three promising avenues towards this goal: the application of plasma enhanced CVD (PECVD) processes, the use of bimetal catalysts, and the choice of hydrocarbon precursor. Despite these efforts, control of graphene layer number and film connectivity remains a significant challenge as reaction temperatures and catalyst thicknesses are reduced [6]. For example, PECVD techniques relying on ionization of the carbon precursor to reduce the energy required for graphene synthesis have yielded quality graphene at 600 °C on predominantly copper Cu/Ni alloys, however incomplete dehydrogenation and multilayer formation is observed upon further temperature reduction due to the reduced catalytic activity of the primarily Cu substrate [14]. Similarly, transition metals with partially filled D orbitals (Fe, Co, Ni) have been identified as suitable candidates for CVD synthesis temperature reduction due to their increased ability for carbon ion stabilization. However, the increased carbon solubility in these metals leads to uncontrollable layer formation upon cooling [15,16,17,18,19]. In attempts to alleviate this issue, Ni has been combined with less catalytic metals, such as Au, to suppress the formation of multilayer films. These catalysts show the potential to enable the formation of few-to-monolayer graphene films at 450 °C, following a 600 °C anneal of the catalyst prior to growth [20]. Though these results are promising, they require catalyst thicknesses of 500 nm or greater to minimize multilayer formation, in addition to the aforementioned catalyst pretreatments at elevated temperatures. In addition to the research efforts mentioned above, numerous gaseous carbon precursors, including methane, ethane, and propane have been investigated. It was found that larger carbon precursor molecules allow graphene synthesis at reduced temperatures due to increased ion stability and reduced energy requirements for dehydrogenation [21,22]. This trend has led to the development of CVD techniques employing solid phase and liquid phase carbon sources to further reduce required reaction temperatures for graphene synthesis through a significant increase in carbon precursor size [23,24,25]. Graphene synthesis at 300 °C has been performed with benzene and poly(methyl methacrylate) (PMMA) on Cu substrates, however 1000 °C pretreatment of the catalyst is required prior to the synthesis [24,26]. These results suggest the importance of the synergistic relationships among the carbon precursor molecule size, the ionization state, the target substrate reactivity, and the carbon solubility and thickness of the catalyst. Although graphene formation on low-reactivity catalysts has been carried out through ionization of the hydrocarbon precursor, and graphene growth on high-reactivity catalysts has been achieved through both bimetal catalysts and increased hydrocarbon precursor sizes, techniques for in situ manipulation of carbon precursors tailored to the specific target substrate have not been thoroughly investigated.

This report demonstrates a unique methodology to gain in-depth understanding of the synergistic relationships between critical growth parameters. This investigation was carried out using a PECVD synthesis technique in which the size and ionization state of carbon precursor molecules reaching the growth catalyst is manipulated to reduce the rate of nucleation and absorption into the catalyst bulk, resulting in the formation of a continuous few-to-monolayer graphene film at 500 °C. This is achieved through control of the inlet between a remote inductively coupled plasma (ICP) location and the catalyst location that enables both ion screening and secondary capacitively coupled plasma (CCP) generation. This precursor screening technique is demonstrated on a 50 nm thick Ni/Cu catalyst (2 wt% Cu), representing a 10-fold reduction in catalyst thickness compared to previously published results, while eliminating the elevated temperature pre-growth anneal required by previous reports [20]. Characterization of the generated plasma species is performed via UV-Vis inspection, while mass spectrometer (MS) characterization of the growth chamber coupled with current monitoring at the catalyst location enables identification of species reaching the catalyst. It is observed that the layer number and defect concentrations can be controlled via ion screening processes, while a secondary ionization procedure leads to further reduction in both defect concentrations and multilayer portions of the film.

## 2. Materials and Methods

### 2.1. Reactor Configuration and Capabilities

**Figure 1:**
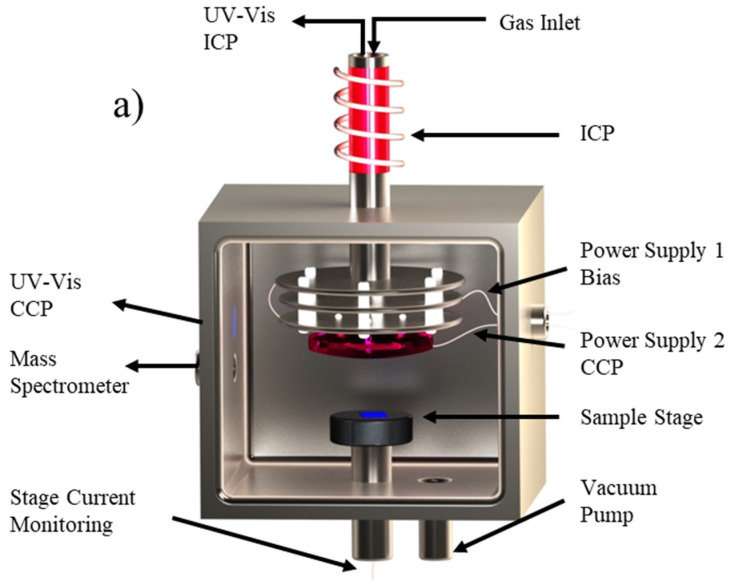

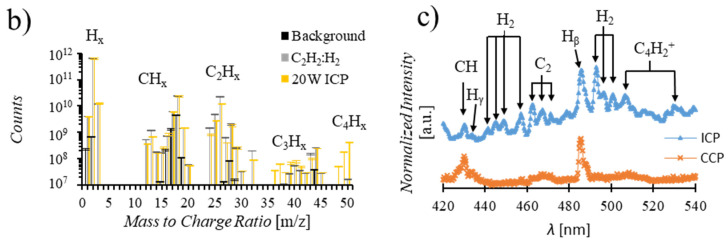
(**a**) Schematic of custom plasma enhanced CVD (PECVD) system with configurable inlet enabling ion screening and secondary plasma generation as well as stage current monitoring, mass spectrometry, and UV-Vis spectroscopy. (**b**) Mass spectrum indicating chamber background (in black) composition is primarily H_2_O and CO_2_ (*m/z* 18, 28, 44). Gas introduction, C_2_H_2_:H_2_ in a 0.1:15 ratio (displayed in grey), results in increased detection of 1 and 2 carbon containing species (*m/z* 13–16, 24–26) while 20W inductively coupled plasma (ICP) ignition (displayed in yellow) results in the detection of 3 and 4 carbon species (*m/z* 36–39, 47–50). (**c**) UV-Vis spectrum collected for a 20W ICP (shown in blue) and a 2.5W capacitively coupled plasma (CCP) (shown in orange) indicate the increased diversity of both hydrogen and carbon signals present at the higher powered ICP while primarily H_β_ and CH ionization events occur in the low power CCP.

**Figure 2:**
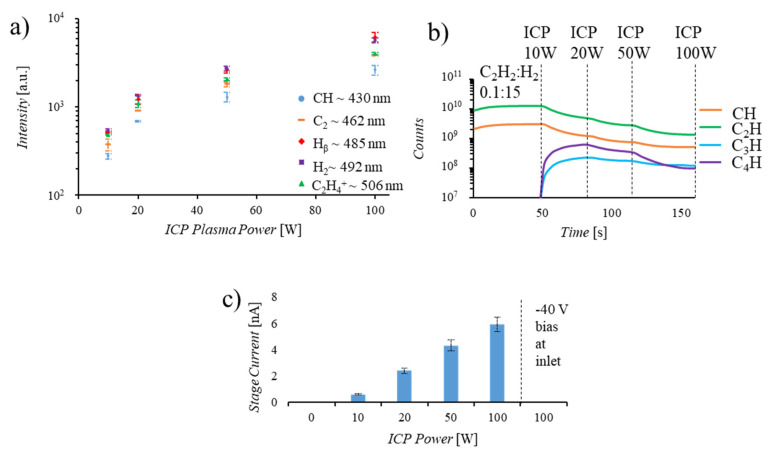
(**a**) UV-Vis spectrum at varied ICP powers indicate that increasing plasma power results in an increased occurrence of ionization events for all species. (**b**) Mass spectrum displaying the increase of 3 and 4 carbon species with plasma ignition and the reduction of all detected species as plasma power increases, indicating that fewer neutral species are reaching the mass spectrometer (MS) detector as ICP power is increased. (**c**) Stage current readings displaying an increase in stage current as ICP power is increased and zero current detected when a screening bias is applied at the chamber inlet, displaying effective reduction of charged species reaching the stage.

All experimental results were obtained in custom-built reactor as shown in Figure 1a, with remote ICP location and configurable inlet along the path from the plasma to the catalyst. A positive or negative voltage can be applied to the inlet plates independently to screen ions and/or generate a secondary CCP. Current monitoring at the sample stage enables characterization of the inlet plate effects on charged species reaching the catalyst. Monitoring of the growth chamber via mass spectrometry enables identification of neutral species reaching the catalyst location through analysis of fragments generated upon ionization at the detector. Ionized species generated in the plasma are not expected to reach the MS which is separated from the main chamber by a leak valve. This is verified by a lack of signal detected when the ionizing component of the MS is turned off in the presence of plasma at the ICP or CCP location. As depicted in Figure 1b, the background composition of the chamber at 1 × 10^−7^ torr is primarily H_2_O and CO_2_. Introduction of C_2_H_2_ and H_2_ results in an expected increase in 1 and 2 carbon species while ignition of a 20W plasma at the remote ICP location results in the generation of 3 and 4 carbon species, in agreement with previously reported characterizations of acetylene plasmas [27,28,29]. Figure 1c displays the UV-Vis spectrum collected at the ICP and CCP locations, confirming the generation of these larger hydrocarbon molecules with the presence of a plasma. Characterization of gaseous species generated both at the remote ICP location and those that reach the mass spectrometer reveal that there is an increase in ionization events (Figure 2a), and a reduction in neutral species reaching the MS detector (Figure 2b) with increasing remote plasma power. However, plasma power variation alone does not enable selection for carbon precursor size as increasing power increases the generation of both large and small species. Additionally, current measurements at the catalyst location during remote plasma operation confirm that primarily positive ionic species are reaching the catalyst and that the application of a negative bias to a reaction chamber inlet plate effectively blocks these ions from reaching the catalyst (Figure 2c). This characterization indicates that, while increasing remote plasma power alone does not enable significant selectivity for the size of species generated, the average size of carbon precursors reaching the catalyst can be increased through remote plasma operation coupled with screening of high energy ions through the application of a negative bias at a chamber inlet plate. The novel design of the reaction chamber enables characterization and manipulation of gaseous species during graphene synthesis, revealing the synergistic relationship between growth parameters.

### 2.2. Reaction Chamber Characterization

UV-Vis characterization was performed through spectrum collection (USB200+, Ocean Insight, Rochester, NY, USA) of ICP and CCP signals through isolated viewports, above the ICP and on the main chamber for CCP. Stage current characterization was performed through Pico ammeter (Keithley 485, Tektronix INC., Beaverton, OR, USA) monitoring of the sample stage. Mass spectrometry (PrismaPro QMG 250 M2, Pfeiffer Vacuum, Nasuhua, NH, USA) was collected in a secondary chamber with differential pumping to maintain 1 × 10^−6^ torr which is connected to the main chamber through a leak valve.

### 2.3. Catalyst Deposition and Graphene Synthesis

50 nm Ni/Cu catalysts were deposited on Si/SiO_2_ wafers through magnetron sputtering (AXXIS, Kurt J. Lesker Company, Jefferson Hills, PA, USA) of 48 nm Ni followed by 2 nm Cu without breaking vacuum. This catalyst composition and thickness was identified through preliminary experimentation to minimize catalyst dewetting during synthesis, observed as thickness is reduced, and enable graphene formation, difficult with increased Cu concentrations, without significant multilayer formation, common with reduced Cu concentrations [30,31]. Graphene synthesis was performed in the custom PECVD chamber initiated by chamber evacuation to base pressure of 1 × 10^−7^ torr followed by heating to 500 °C under 15 sccm of H_2_, resulting in a chamber pressure of 50 mTorr. To promote cleaning and alloying of the catalyst, the 1 cm × 1 cm sample was held at 500 °C for 2 min under H_2_ flow prior to introduction of the hydrocarbon precursor. Graphene growth was initiated by introduction of C_2_H_2_ at 0.1 sccm and ignition of a 20W ICP plasma for 1 min. Screening bias and secondary CCP were applied according to the desired synthesis regime through a −40 V bias application (PSFX, XP Glassman, High Bridge, NJ, USA) to the first inlet plate or CCP generation at 2.5W (Bertan 205A, Spellman HVEC, Hauppauge, NY, USA) with a negative bias applied to the second plate. After preliminary experimentation, a −40 V screening bias was identified as optimal to stop all detection of current at the sample location without plasma ignition or arcing at the screening location during the synthesis processes. Following completion of the synthesis regime, ICP, CCP, and screening bias powers were set to zero, as well as the C_2_H_2_ flow rate. Finally, the sample was allowed to cool under 15 sccm H_2_ until 150 °C over approximately 15 min before venting the chamber to atmosphere.

### 2.4. Graphene Transfer and Characterization

Graphene was transferred from the catalyst through spin coating (WS-650, Laurell Technologies, North Wales, PA, USA) 300 nm polymethyl methacrylate (PMMA) support and baking in air at 150 °C for 5 min. The sample was submerged in 0.5 M FeCl_3_ to etch both Ni and Cu until the graphene/PMMA floated to the surface. Following 5 rinses for 1 min each in DI water, the graphene with PMMA support was transferred to fresh Si/SiO_2_ and PMMA was removed in acetone. Raman characterization was performed on a Jobin Yvon HR800 (HORIBA, Kyoto, Japan) with 532 nm laser excitation and mapping acquisition capabilities through a motorized sample stage. Raman map characterization and spectrum averaging were performed through in-house software, written in R, to peak fit D, G, and 2D bands for each spectrum collected and generate 2D plots.

## 3. Results and Discussion

**Figure 3:**
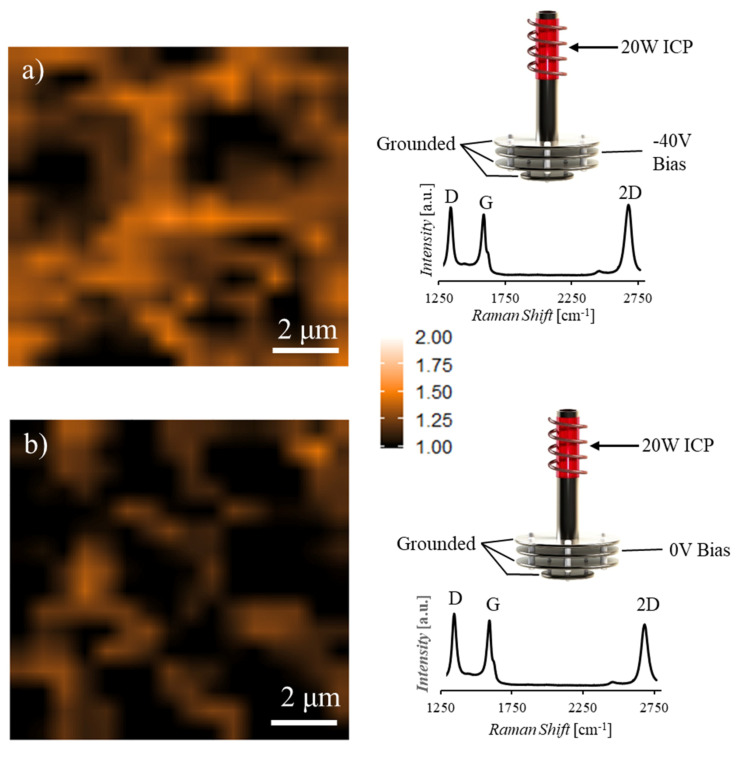
Ion screening bias effects on graphene film layer number. 100 µm^2^ Raman I_2D/G_ maps and accompanying average Raman spectrum over the mapped area for graphene samples synthesized with 20W ICP and (**a**) −40V screening bias applied at the chamber inlet and (**b**) no applied screening bias during the 1-min synthesis. Increased multilayer formation (black portions of the mapped area) is observed on the unscreened case when compared to the biased case while both average Raman spectrums indicate elevated defect concentrations, with average I_D/G_ > 1.

To identify the effects of in situ precursor manipulation on achievable graphene quality, all reported synthesis is performed as described in Section 2.3 with only variations of the plasma generation location and energized state of the screening plate. Following transfer of the graphene films, Raman mapping is performed to characterize quality and uniformity with ratios of the intensity of D, G, and 2D bands as well as the full width at half maximum (FWHM) of the 2D peak to determine the layer number and defect density of the films. Fewer layers are present with increasing I_2D/G_, and defect densities increase with increasing I_D/G_. While pristine monolayer graphene displays a nearly undetectable I_D/G_ and an I_2D/G_ ≥ 2, when defects are present monolayer graphene is identified by an I_2D/G_ > 1 and FWHM_2D_ < 100 cm^−1^ [32,33]. To categorize areas of multilayer and monolayer graphene in these samples, 2D maps of I_2D/G_ are presented with color scales fixed between 1 and 2, with black areas, I_2D/G_ ≤ 1, representing multilayer portions of the film, white areas, I_2D/G_ ≥ 2, representing low defect density monolayer portions of the film, and orange areas, 1 < I_2D/G_ < 2, representing few-to-monolayer portions of the film. Figure 3a,b display 100 μm^2^ I_2D/G_ Raman maps, with accompanying average Raman spectra for the mapped areas, of samples synthesized with and without an applied screening bias at the inlet plate, respectively. It is observed that with the application of a screening bias, both average layer number and areas of multilayer (areas with I_2D/G_ ≤ 1 indicated by black portions of the Raman map) are reduced compared to the unscreened case by 62%. The reduction of multilayer portions of the film under the applied bias condition is attributed to the screening of high energy ions that are more readily dehydrogenated and adsorbed into the catalyst bulk, leading to rapid saturation and multilayer formation upon cooling. While these ions are screened by the applied bias, the neutral molecules, including 3 and 4 carbon species (*m/z* 36–39, 47–50) generated in the remote plasma, are permitted to reach the catalyst location and participate in graphene formation at the catalyst surface. Though a significant reduction in multilayer portions is observed, the graphene film remains highly defective. The films (Figure 3a,b) have an average I_D/G_ of 1.2, with an increased background between the D and G peaks indicative of remaining sp^3^ hybridization through C-H bonds [34,35].

**Figure 4:**
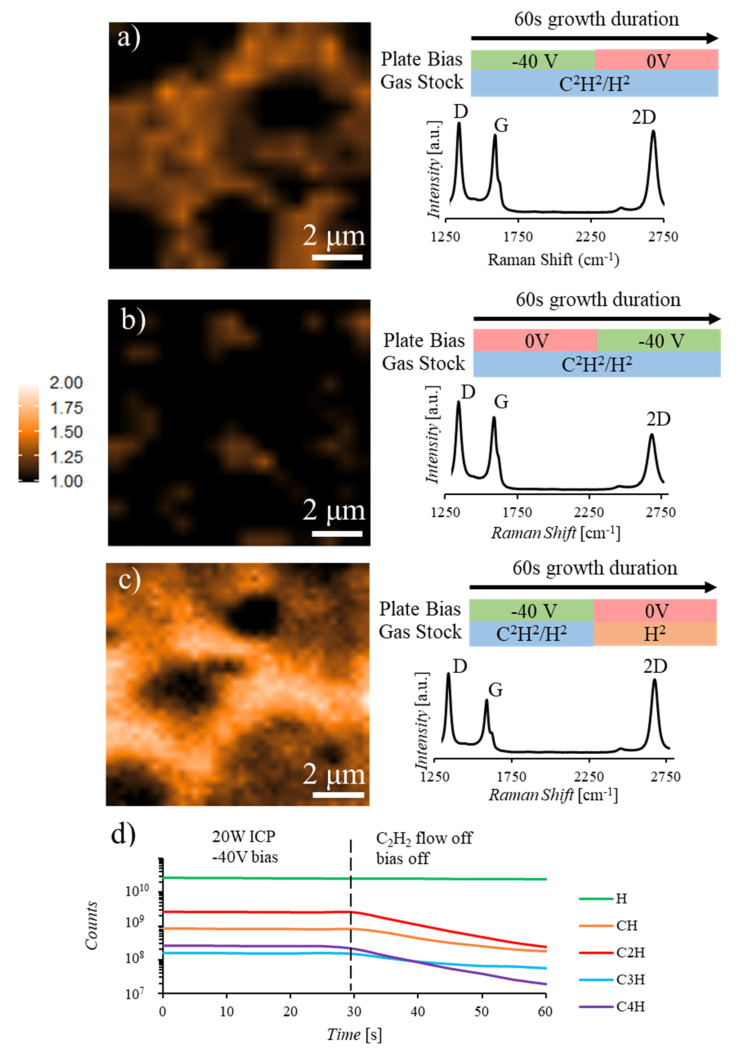
Multistage graphene synthesis with ion screening bias application and carbon precursor presence at differing portions of the growth. 100 µm^2^ Raman I_2D/G_ maps and accompanying average Raman spectrum over the mapped area for graphene samples synthesized with 20W ICP for 1 min and (**a**) −40V screening bias for the first 30 s of synthesis, (**b**) −40V screening bias applied for the second 30 s of synthesis, and (**c**) −40V screening bias applied for the first 30 s of synthesis and both the bias and C_2_H_2_ feedstock removed for the second 30 s of synthesis. An increased presence of multilayer portions of the film is observed in (**b**) compared to (**a**) while in (**c**) a significant reduction in multilayer is detected compared to the other two cases. (**d**) Mass spectrum data for the synthesis in (**c**) displaying a reduction in 1, 2, 3, and 4 carbon species with removal of acetylene from the feedstock to the ICP generation location.

Synthesis results under the biased plate condition indicate that to reduce the layer number and defect densities of the graphene films, both a reduction in nucleation density and an increase in dehydrogenation rates must be achieved. To characterize the capability of this ion screening technique toward achieving these goals, multistage growths were performed in which the screening bias was applied for a portion of the synthesis. Figure 4a,b display Raman maps and accompanying average Raman spectra from samples in which the bias was applied for the first or second half of the 1-min synthesis, respectively. The synthesis performed with a screening bias for the first 30 s of the growth (Figure 4a) displays a small increase in multilayer coverage when compared to the synthesis with bias application for the growth in its entirety (Figure 3a). This result indicates that the initial screening of high energy ions results in nucleation occurring primarily from neutral and larger carbon containing species and the removal of the screening bias allows high energy ions to reach the catalyst and continue both growth at the surface and saturation of the catalyst bulk. Conversely, the sample produced with a screening bias applied for the second 30 s (Figure 4b) displays a significant increase in multilayer formation indicating high rates of nucleation, growth, and absorption into the catalyst bulk during the initial 30 s where no screening bias is applied. Application of the screening bias during the final 30 s of the synthesis removes the ionized species responsible for dehydrogenation and film completion, resulting in increased multilayer formation. Further reduction in multilayer portions of the film and defect density (Figure 4c) is achieved through application of the bias for the first 30 s of the synthesis followed by removal of both the bias and the carbon precursor feed stock to the remote plasma location for the second half of the synthesis (Figure 4d). This results in reduced nucleation rates during the initial stage of the growth, associated with bias application, and, with the removal of both the bias and the carbon feedstock, increased rates of dehydrogenation without continued layer formation during the second half of the synthesis. This multistage ion screening synthesis technique enables production of continuous and predominantly few-to-monolayer, 91% I_2D/G_ > 1, graphene at 500 °C without requiring an increased temperature anneal.

**Figure 5:**
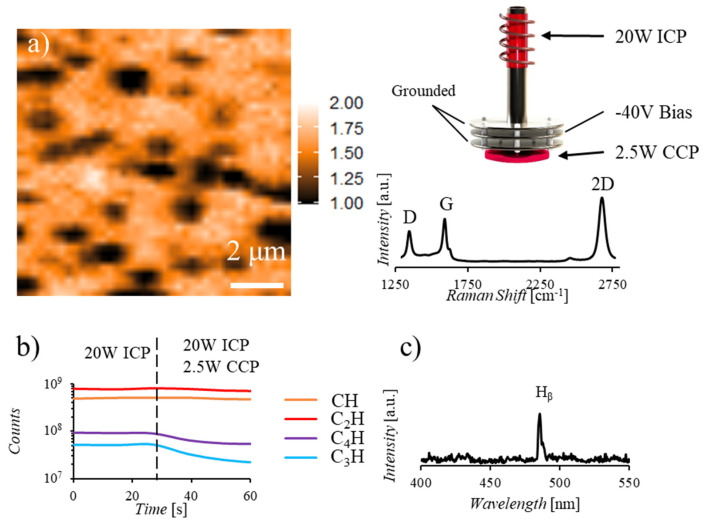
Graphene synthesis with both ICP and secondary CCP resulting in reduced layer number and defect density. Raman I_2D/G_ map, (**a**), indicating primarily monolayer formation (95% I_2D/G_ > 1) and accompanying average Raman spectrum displaying reduced defect densities compared to multistage synthesis results in Figure 4. (**b**) Mass spectrum depicting the change in hydrocarbon species present with the ignition of a secondary CCP. Note that the number of 3 and 4 carbon species is reduced with ignition of the secondary plasma while the number of 1 and 2 carbon species remains nearly constant. (**c**) UV-Vis spectrum of CCP collected while ICP plasma generation is also occurring, indicating primarily H ionization. Note the reduction in CH and C_2_ ionization events compared to the CCP spectrum, Figure 1c, collected when no upstream ICP is present.

Further control over the reactivity of species reaching the catalyst location can be achieved through the generation of a low power, 2.5 W, secondary plasma after the ion screening location. Figure 5a shows a Raman map and average Raman spectrum of graphene produced during a 1-min synthesis with both a remote plasma and a secondary plasma, representing a significant reduction in average defect densities, from 1.4 to 0.7 I_D/G_, while increasing few-to-monolayer coverage, 95% I_2D/G_ > 1. MS characterization (Figure 5b) of the reaction environment reveals a reduction in 3 and 4 carbon species with the ignition of a secondary plasma while the concentration of 1 and 2 carbon species remains relatively unaffected. Additionally, UV-Vis monitoring of the secondary CCP (Figure 5c) reveals that primarily H ionization events occur when the remote ICP is present while both H and CH ionization events occur when only the secondary CCP is present (Figure 1c). These results, coupled with the detection of a current at the sample location upon ignition of the secondary CCP, indicate that 3 and 4 carbon species generated in the 20W ICP are not reaching the MS and may be the primary species ionized at the secondary CCP location prior to interacting with the catalyst. Comparing the Raman map under this two-plasma, ICP and CCP, condition (Figure 5a) to the map of the sample synthesized under a multistage bias condition (Figure 4c), an increased number but decreased size of multilayer islands is observed in the two-plasma case. We hypothesize that this phenomenon results from an increased nucleation rate associated with larger carbon precursors which are generated at the ICP location and ionized at the CCP location before reaching the catalyst. These larger ionized species are more likely to nucleate at the catalyst surface, resulting in the increased number of multilayer islands observed, but are less likely to be absorbed into the catalyst bulk, resulting in the overall increase in few-to-monolayer content of the film. While bias application alone screens high energy ions and a multistage bias synthesis condition reduces multilayer formation, this secondary ionization technique increases few-to-monolayer coverage to 95% through both increasing the reactivity of carbon precursors and reducing the rate of catalyst saturation.

This phenomenon of controlling the concentration and ionization states of precursor molecules permitted to interact with the growth substrate has resulted in the significant increase in few-to-monolayer coverage in the secondary bias case. While the dependence on carbon species size and ionization state has been demonstrated, the specific roles of each ionized species within the larger groups, i.e., 3 carbon and 4 carbon species, will require in situ characterization of reactions occurring at the catalysts surface. Future work in this area should lead to improvements in targeting specific precursor species to intended substrates and continue to advance efforts toward graphene inclusion in a variety of fields.

## 4. Conclusions

In summary, we have demonstrated graphene synthesis techniques utilizing in situ manipulation of carbon precursors generated during plasma enhanced chemical vapor deposition to achieve continuous graphene films at reduced temperatures on reduced catalyst thicknesses. This experimental approach has allowed us to gain an in-depth understanding of the correlation among the parameters investigated. Moreover, this synthesis technique, which is not represented in literature, enables the manipulation of nucleation density, layer number, and defect densities though control of carbon precursor sizes and ionization states. Screening bias application between a remote ionization location and the sample location facilitates targeting of larger neutral molecules while a secondary ionization event can increase the reactivity of these molecules. Our results demonstrate that by utilizing this technique a few-to-monolayer graphene (with average Raman D to G peak intensity ratio I_D/G_ = 0.7) can be synthesized on 50 nm Ni/Cu thin film catalysts at 500 °C, without the need for any high temperature catalyst pretreatments. This technique represents not only an avenue for continued reduction to synthesis temperature and transition metal catalysts thickness requirements but reveals a novel method for active species control in broader PECVD synthesis techniques.
